# Role of Oral Microbiota Dysbiosis in the Development and Progression of Oral Lichen Planus

**DOI:** 10.3390/jpm14040386

**Published:** 2024-04-03

**Authors:** Alessandro Lavoro, Giovanni Cultrera, Giuseppe Gattuso, Cinzia Lombardo, Luca Falzone, Candido Saverio, Massimo Libra, Mario Salmeri

**Affiliations:** 1 Department of Biomedical and Biotechnological Sciences, University of Catania, Via Santa Sofia 97, 95123 Catania, Italy; alessandro.lavoro@unict.it (A.L.); giovanni.cultrera@studium.unict.it (G.C.); giuseppe.gattuso@phd.unict.it (G.G.); cinzia.lombardo@phd.unict.it (C.L.); scandido@unict.it (C.S.); m.libra@unict.it (M.L.); m.salmeri@unict.it (M.S.); 2 Research Center for Prevention, Diagnosis and Treatment of Cancer, University of Catania, 95123 Catania, Italy

**Keywords:** oral microbiota, oral lichen planus, autoimmune disease, oral dysbiosis, oral diseases, oral cavity, inflammation, biomarker, squamous-cell carcinoma

## Abstract

Oral lichen planus (OLP) is a chronic inflammatory autoimmune disease of the oral cavity with malignant potential affecting 1.01% of the worldwide population. The clinical patterns of this oral disorder, characterized by relapses and remissions of the lesions, appear on buccal, lingual, gingival, and labial mucosa causing a significant reduction in the quality of life. Currently, there are no specific treatments for this disease, and the available therapies with topical and systemic corticosteroids only reduce symptoms. Although the etiopathogenesis of this pathological condition has not been completely understood yet, several exogenous and endogenous risk factors have been proposed over the years. The present review article summarized the underlying mechanisms of action involved in the onset of OLP and the most well-known triggering factors. According to the current data, oral microbiota dysbiosis could represent a potential diagnostic biomarker for OLP. However, further studies should be undertaken to validate their use in clinical practice, as well as to provide a better understanding of mechanisms of action and develop novel effective intervention strategies against OLP.

## 1. Introduction

Lichen planus, from the Greek “*leichen*” (tree moss) and the Latin “*planus*” (flat), was first described by Erasmus Wilson in the second half of the nineteenth century [1]. It represents a chronic inflammatory mucocutaneous disease characterized by polygonal, pruritic, and flat-topped papules with several localizations [2,3]. As reported in the literature, lichen planus affects 0.1–4% of the worldwide population, with most cases occurring among middle-aged people of both sexes (range: 30–60 years). However, some studies have also described this pathological condition in childhood and its association with other oral lesions [4,5]. Although it is considered an autoimmune disease related to the cell-mediated damage of the basal keratinocytes, the exact etiology of lichen planus remains unknown [6].

The skin and the oral mucosa represent the main affected zones, but other cutaneous sites, such as the hair, scalp, and nails, as well as the esophagus, genitalia, and conjunctiva, can be involved [7,8,9]. The oral form, also known as oral lichen planus (OLP), is one of the most common autoimmune disorders of the oral mucosa [10]. It is generally characterized by bilateral and symmetrical lesions that might progress into very painful erosions, causing a significant reduction in the quality of life [11]. In the oral cavity, the most affected region is the buccal mucosa, followed by the lingual, gingival, and labial mucosa [10,12].

According to Andreasen’s classification [13], it is possible to distinguish six clinical forms of OLP (reticular, erosive, atrophic, plaque-like, bullous, and papular). These subtypes can change their clinical morphology and can occur simultaneously [13].

Although the etiology and pathogenesis of OLP have not been completely understood yet, several endogenous and exogenous factors have been proposed as triggering factors, including changes in the composition of oral microbiota [14,15]. The oral microbiota, identified for the first time in 1670 by Dutchman Antony van Leeuwenhoek, is composed of several species of microorganisms (~700 species), and this observation was recently confirmed [16,17]. Interestingly, bacteria, fungi, viruses, archaea, and protozoa create different microenvironments in the oral cavity, such as the tongue, teeth, hard and soft palate, gingival sulcus, and tonsils [18,19].

Over the years, an increasing number of studies have demonstrated that oral microbiota plays a key role in ensuring the maintenance of systemic and oral health [20,21,22,23]. Of note, the imbalance of oral microbiota may cause several oral infectious diseases, including periodontitis and dental caries, as well as oral mucosal disorders [24,25]. In addition, the dysbiosis of oral microbiota is also associated with various systemic diseases, such as pneumonia, heart diseases, and pancreatic and esophageal cancers [26,27,28,29]. Since the dysbiosis of oral microbiota is involved in the development of several diseases, it has recently gained the attention of the scientific community. Therefore, the present review article aims to report a summary of the studies that have been conducted to date to provide a better understanding of the potential relationship existing between the alteration of oral microbiota and the onset of OLP.

## 2. OLP: Epidemiology and Clinical Variants

OLP is defined as a chronic inflammatory autoimmune disease of the oral cavity affecting 1.01% of the global population, with the highest incidence in Europe (1.43%) and the lowest in India (0.49%) [30]. This oral lesion, characterized by relapses and remissions, mainly occurs in females rather than in males (ratio 2:1), and the prevalence in both sexes significantly increases after 40 years old [31].

It is possible to distinguish six clinical patterns of OLP that can be found individually or in combination. Reticular OLP represents the most common clinical type and appears with white papules, also known as “Wickham striae”, surrounded by an erythematous area. Reticular lesions are generally asymptomatic and can affect the buccal mucosa, tongue, gingiva, palate, or lips with a bilateral and symmetrical localization [32]. On the contrary, erosive OLP is a variant characterized by symptomatic lesions ranging from a burning sensation to severe pain. Clinically, the border of erosive lesions is outlined by keratotic white striae, and the affected areas (gingiva and tongue) show ulcerations generally covered by a fibrin plaque or a pseudomembrane. Moreover, these lesions can simultaneously occur with the reticular ones [33]. Similarly, patients affected by the atrophic form generally report pain when chewing and speaking and mucosal atrophy. Of note, these red diffuse lesions usually involve the gingiva or the dorsum of the tongue and may be confused with the reticular type due to the presence of an erythematous area. However, atrophic lesions hardly heal spontaneously [34].

The plaque-like form of OLP appears with whitish and raised lesions, and it is similar to oral leukoplakia. This clinical variant mainly affects tobacco smokers in which the most frequently interested areas are the tongue and buccal mucosa [35]. Finally, bullous and papular subtypes are the least common clinical forms. Regarding the bullous type, the lesions consist of ulcerated and painful areas due to the rupture of bullae, while the papular form is characterized by white papules ranging from 0.5 to 1.0 mm in diameter generally in combination with other variants. In both cases, lesions may occur in the soft palate or lips [36]. The clinical variants of OLP and their clinical characteristics are summarized in Table 1.

Despite all these forms of OLP, a clear distinction should be made between OLP and oral lichenoids. In particular, these two lesions are distinct conditions that affect the oral mucosa, sharing some similarities but also exhibiting significant differences in their etiology, clinical presentation, histopathology, prognosis, and treatment approach.

Oral lichenoids are usually driven by an inflammatory reaction to several agents including medications, dental materials like amalgam fillings, and specific oral hygiene products. These lesions often manifest as white patches or plaques with or without surrounding erythema and can occur bilaterally on the buccal mucosa, tongue, gingiva, or palate. Histologically, oral lichenoid displays features resembling OLP but may exhibit more pronounced inflammation, damage to the basal cell layer, and neoplastic potential, along with the presence of Civatte bodies [37].

While oral lichenoid is often associated with identifiable trigger factors and external agents, OLP is not typically linked to specific triggers and is considered primarily autoimmune in nature. The management of lichenoids involves identifying and eliminating potential triggers, along with symptomatic treatment using topical corticosteroids or other immunomodulatory agents. Conversely, the treatment of OLP aims to alleviate symptoms and control inflammation, with topical corticosteroids being the mainstay of therapy and systemic immunosuppressive agents reserved for severe or refractory cases [38].

## 3. Etiopathogenesis of OLP

Although the etiology of OLP is not completely understood, several triggering factors have been suggested to be involved in the onset of this oral disease. For example, many studies have documented a positive correlation between OLP and the *Hepatitis C virus* (*HCV*). This association is probably due to the capability of *HCV* to replicate in oral mucosa cells and its high variability [39,40,41]. Besides HCV, other viral infections seem to be implicated as etiological factors of OLP, including *Human papillomavirus* (*HPV*), *Cytomegalovirus* (*CMV*), *Epstein–Barr virus* (*EBV*), and *Herpes simplex virus* (*HSV*) [42,43,44].

Interestingly, medications (anti-hypertensive, hypoglycemic, anti-inflammatory, and anti-malarial drugs) and dental restoration materials (amalgam, metallic mercury, and nickel) have been proposed as exogenous factors [45,46]. Regarding endogenous factors, some studies have focused on the involvement of genetic factors. Specifically, genetic polymorphisms of different cytokines, such as *Interferon γ* (*INF-γ*) and *Tumor necrosis factor α* (*TNF-α*), may play an important role in the development of OLP [47]. However, further studies are needed to confirm whether this oral disease is genetically determined. At the same time, psychological disorders have also been investigated as triggering factors. Despite the controversy over the involvement of these factors, some studies have observed higher levels of anxiety, stress, and depression in patients affected by OLP compared to healthy subjects [48,49].

Similar to the etiology, the pathogenesis of OLP has not yet been fully understood. Over the years, antigen-specific and non-specific mechanisms have been described to elucidate the pathogenesis of this disease [50,51]. As reported in the literature, a cell-mediated immune response could induce the apoptosis of keratinocytes. According to this hypothesis, the activation of CD8^+^ cytotoxic T lymphocytes (CD8^+^ T cells) is determined after their direct migration in the epithelium, through the antigen expressed by major histocompatibility complex class I (MHC class I), or indirectly, through the activation of CD4^+^ helper T lymphocytes (CD4^+^ T cells). Interestingly, Langerhans cells seem to play a key role in the activation of CD4^+^ T cells because they present the responsible antigen via MHC class II molecules. At this point, activated CD8^+^ T cells can induce the apoptosis of basal keratinocytes through TNF-α or a Fas–Fas ligand mechanism [52].

Another mechanism that may play an important role in the pathogenesis of this oral disease is represented by the activation of matrix metalloproteinases (MMPs). Of note, some studies have highlighted that the expression levels of *MMP-9* and *MMP-2* were higher in OLP patients compared to healthy controls, suggesting that non-specific mechanisms may be also responsible for the pathogenesis [53,54]. Moreover, RANTES (Regulated on Activation, Normal T cell Expressed and Secreted) could have an important role in the chronic course of this disease. In this regard, Zhao and colleagues [55] reported that this pro-inflammatory cytokine, secreted by several cell types including activated T lymphocytes and keratinocytes, induced the degranulation of mast cells.

## 4. OLP Diagnosis, Treatment, and Malignant Potential

Over the years, there have been many attempts to provide a specific definition of OLP. For example, the World Health Organization (WHO) published the clinical and histological diagnostic criteria of OLP in 1978 [56], and subsequently, some modifications were proposed. Of note, the specific histopathological criteria for OLP, introduced by van der Meij and van der Waal in 2003 [57], were as follows: the presence of a well-defined, band-like zone of cellular infiltration that is confined to the superficial part of the connective tissue mainly consisting of T lymphocytes; signs of liquefaction degeneration in the basal cell layer; and the absence of epithelial dysplasia. Recently, a new set of diagnostic criteria was proposed by the American Academy of Oral and Maxillofacial Pathology (AAOMP) in 2016. As reported by Indrees et al. [58], this new proposal obtained more success among pathologists than the previous criteria, but the issue has not yet been resolved.

Although the diagnosis should be based on clinical and histological examination, in classical lesions, the clinical appearance may be enough to establish a diagnosis of OLP [6]. However, an oral biopsy with histological findings still represents the gold standard method to confirm the clinical diagnosis. More recently, the use of liquid biopsy has also been proposed. In addition, direct immunofluorescence has been described as an auxiliary diagnostic tool useful for cases with similar characteristics to other diseases [59]. Among the proposed biomarkers for the diagnosis of OLP and other oral lesions, including cancer, there are also microRNAs and other non-coding RNAs; however, further validation experiments are needed to clearly elucidate their diagnostic role [60,61]

Recently, liquid biopsy has attracted great interest in the early diagnosis of OLP and other oral lesions [62]. Specifically, liquid biopsy offers several advantages compared to conventional tissue biopsies, including minimal invasiveness, the repeatability of the sampling, and the ability to evaluate dynamic changes in disease progression and biomarker alterations, including microbial changes [59].

Recent studies have demonstrated the application of liquid biopsy for the diagnosis and monitoring of OLP. For example, Gissi DB and colleagues (2019) identified DNA methylation alterations associated with the development of OLP by analyzing oral brush samples and achieving high diagnostic accuracy compared to traditional methods suggesting that similar results could be achieved by analyzing saliva samples [63]. Similarly, Byun J.S. et al. (2015) investigated the feasibility of using salivary exosomal microRNAs as biomarkers for OLP diagnosis, revealing promising results in discriminating OLP patients from healthy controls [64].

Moreover, liquid biopsy holds promise for evaluating alterations in oral microbiota and microbiome composition associated with OLP and other oral lesions. By analyzing microbial DNA and RNA present in saliva, researchers can identify microbial signatures indicative of disease status and dysbiosis. For instance, Zhong E.F. et al. (2020) employed next-generation sequencing for the analysis of saliva samples identifying specific microbial community changes in OLP patients, revealing distinct microbiome profiles compared to healthy individuals [65]. Similarly, a study by Bankvall M et al. (2022) utilized metagenomic sequencing to identify specific bacterial taxa associated with OLP pathogenesis, highlighting the potential of liquid biopsy in elucidating microbial dysbiosis in oral diseases [66].

As regards the therapeutic approaches for this lesion, currently, there is no effective therapy for OLP, and its management can be difficult. In general, the asymptomatic reticular lesions require only monitoring and no therapy, while atrophic and erosive forms are generally treated to alleviate the symptoms [67]. Specifically, the first-line treatment is represented by topical corticosteroids administered via ointments, such as triamcinolone, fluocinolone acetonide, or clobetasol propionate. In addition, other topical agents, including retinoids and cyclosporine, can be alternatively used for the management of OLP [68]. Second-line therapy is based on systemic corticosteroids, including mycophenolate mofetil or methotrexate, whose administration is effective for the long-term control of symptoms and for extending remission periods [69]. At the same time, good oral hygiene, as well as the daily use of aloe vera mouthwash, a periodic inspection of dental restorations, and plaque control may help to reduce symptoms [70,71]. Regarding conventional surgery, excision is generally used to remove only confirmed dysplastic areas and small lesions [72]. Other therapeutic alternatives are represented by cryotherapy, laser therapy, and Narrowband UVB therapy [73,74,75].

Interestingly, the WHO classified OLP as a potentially pre-cancerous condition in 2005 [76]. Over the years, several studies have focused on the frequency of oral squamous-cell carcinoma among patients affected by this autoimmune disorder and reported an incidence ranging from 0.8% to 4.2% over periods of observation from 6 months to 20 years depending on the study [77,78,79]. In the case of OLP-derived oral cancer, the treatments are completely different based mainly on the use of standard chemotherapy (cisplatin, carboplatin, 5-fluorouracil, paclitaxel, docetaxel, or hydroxyurea) or radiotherapy when surgery is not suitable [80,81,82].

However, it is difficult to establish the malignant potential of this oral disease due to the heterogeneous diagnostic criteria and discontinuous follow-up visits [15,83]. Therefore, further studies are needed to better clarify the involvement of OLP in the onset of oral squamous-cell carcinoma.

## 5. Oral Microbiota Composition and Location

**Figure 1:**
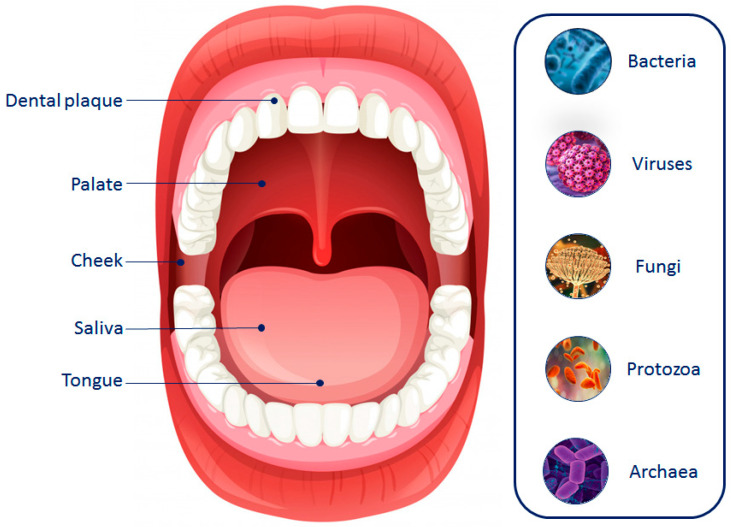
The oral microbiota community and its localization in the oral cavity.

The oral cavity contains the second largest microbiota in the human body, second only to the intestinal one [84]. Specifically, oral microbiota is characterized by a wide spectrum of microorganisms, including bacteria, fungi, archaea, viruses, and protozoa (Figure 1), each of which has a critical role in maintaining the host’s health status [85].

Archaea represent a very small part of the oral microbiota and, although they can be found in healthy subjects, their relative amount is higher in individuals affected by oral disorders. Generally, the most common genera are *Thermoplasmatales*, *Methanobrevibacter*, *Methanobacterium*, and *Methanosphaera* [86,87]. Similarly, the presence of viruses in the oral cavity is related to oral diseases. For example, *HSV* was detected in the oral cavity of children affected by gingivostomatitis [88], while *HPV* was observed in patients with oral lesions, such as oral condyloma [89]. Conversely, fungi have also been observed under physiological conditions as essential components of oral microbiota. Among these, the *Candida* genus represents the most abundant, followed by *Saccharomyces*, *Aspergillus*, and *Cryptococcus* [90]. Bacteria are considered the most abundant group of microorganisms. *Firmicutes*, *Actinobacteria*, *Proteobacteria*, *Fusobacteria*, *Bacteroidetes*, and *Spirochetes* are the six major phyla detected under disease-free conditions and constitute 96% of the oral bacteria community [91]. At the genus level, *Streptococcus* is the most abundant, followed by *Haemophilus*, *Prevotella*, *Neisseria*, and *Fusobacterium* [92].

Interestingly, a description of human-associated oral bacteria characteristics and genetic information is provided by the expanded Human Oral Microbiome Database (eHOMD). Briefly, a total of 774 microbial species are listed in the eHOMD, of which 58% are named, 16% are unnamed but cultivated, and 26% are known only as uncultivated phylotypes due to the limitations of specific culture conditions (nutrients, temperature, pH, and interaction with other microorganisms) (https://www.homd.org/, accessed on 29 September 2023).

Several niches with unique growth conditions can be observed in the oral cavity, including saliva and dental plaque, as well as soft tissues, such as the cheek, soft palate, and tongue (Figure 1) [93]. For example, the main phyla that constitute the microbial community of saliva are represented by *Firmicutes* and *Bacterioidetes*. However, salivary microorganisms have poor long-term stability compared to other oral niches due to their continuous infiltration in tissue surfaces [94]. Conversely, the tooth surface provides a stable microenvironment for the development of dental plaque where Firmicutes and Actinobacteria are the most detected phyla. Of note, since dental plaque (biofilm) can be divided into supra- and subgingival plaque, the microbial profile widely changes depending on the considered location [95,96]. Regarding soft tissues, the cheek and soft palate are only characterized by a bacterial monolayer, while the tongue presents multilayers where *Streptococcus* is the dominant genus [86,97].

Despite the fact that the interest of the scientific community in oral microbiota composition has exponentially grown in the last few years, many controversies remain regarding a specific profile due to several exogenous and endogenous factors that may influence the diversity in each subject [98,99]. For example, Anukam and Agbakoba [100] studied the impact of aging on the composition of oral microbiota and observed substantial differences in the abundance of species among the considered groups. Moreover, in 2018, Galvão-Moreira and colleagues [101] analyzed saliva samples collected from male and female patients (18–40 years old) and noted that there was a significant difference in *Streptococcus mutans* levels probably related to the gender and age of subjects. At the same time, exogenous factors, such as diet or tobacco smoking, have been investigated for their capability to modulate oral microbiota [102,103]. However, the relationship between the aforementioned factors and microbial community composition is not yet completely understood, and further studies are needed to better elucidate the mechanisms of action.

## 6. Oral Microbiota and Oral Diseases

**Figure 2:**
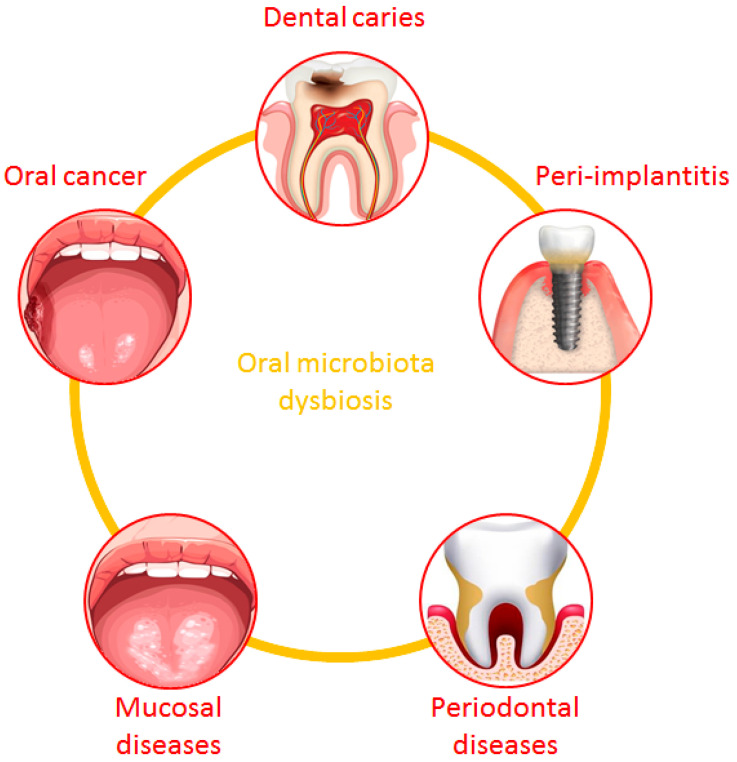
Oral diseases related to the imbalance of oral microbiota.

As described in the literature, oral microbiota plays a key role in maintaining health status. However, its imbalance, characterized by the loss of diversity, the reduction in beneficial microorganisms, and the increase in pathogenic ones, has been widely related to the onset of several oral diseases, such as dental caries, peri-implantitis, periodontal and mucosal diseases, and oral cancer (Figure 2) [104,105,106].

Dental caries is defined as one of the most common non-communicable disorders of the oral cavity occurring at any age. This dynamic disease, which leads to the progressive destruction of dental hard tissues and tooth loss, is due to a wide range of triggering factors, including oral microbiota dysbiosis [107]. Regarding this, in 2019, Vieira and colleagues [108] used saliva samples from caries-free and caries-active children to compare bacteria species of the two groups. Although *Streptococcus* and *Candida* were similar in both cases, the researchers observed that *Rothia mucilaginosa* and *Bacteroides thetaiotaomicron* were more abundant in the caries-active group compared to a caries-free group where *Staphylococcus epidermidis* was enriched [108]. Recently, Zhang and colleagues [109] evaluated the composition of tongue microbiota in primary school children. Of note, they found that the relative abundance of *Fusobacterium periodonticum*, *Porphyromonas pasteri*, and *Neisseria subflava* was higher in children without dental caries [109].

Peri-implantitis is another oral pathological condition affecting the hard and soft tissues surrounding dental implants. It is characterized by inflammation and the progressive loss of supporting bone [110]. Despite the fact that the pathogenesis of peri-implantitis has not yet been well defined, some studies have recently investigated the potential key role of oral microbiota in the onset of this disorder. For example, in 2017, Sainz-Martin and colleagues [111] noted several differences in the microbial composition of peri-implant sites under two clinical conditions. Specifically, *Spirochetes*, *Bacteroidetes*, and *Synergistetes* phyla were more abundant in the affected group, while *Actinobacteria* was higher in the healthy group. At the genus level, *Treponema* and *Porphyromonas* were mainly detected in peri-implantitis, whereas *Neisseria* and *Rothia* characterized non-diseased sites, suggesting the involvement of oral microbiota dysbiosis in the development of this oral disease [105]. Similarly, Apatzidou et al. [112] observed significant differences in the oral microbiota of subjects affected by peri-implantitis and with healthy dental sites. Specifically, the genera *Streptococcus* and *Actinobacillus* were related to health status, while *Porphyromonas* and *Prevotella* were specific for the pathological condition [112].

Periodontal diseases comprise several oral disorders that occur both in adolescents and adults. Among these, gingivitis is considered the most common form, and it is characterized by the inflammation of soft tissues, swelling and bleeding of gums, and pain at the tooth level [113]. Another classic periodontal disease is represented by periodontitis, an inflammatory condition of periodontal tissues mainly found in adults, whose progression in the chronic form leads to the progressive destruction of bone support and tooth loss [114]. Many studies have demonstrated that both these two clinical conditions are related to the bacterial community of the oral cavity. Interestingly, Deng et al. [115] analyzed the subgingival microbial profile of subjects with gingivitis and healthy controls observing a significant difference. Specifically, the genera *Treponema*, *Tannerella*, and *Porphyromonas* showed higher levels in the gingivitis group, while the healthy group was characterized by a higher relative abundance of the *Capnocytophaga* genus [115]. Similarly, Lundmark and colleagues [116] evaluated the potential interaction between salivary microbiota and periodontitis. Briefly, the researchers found that the relative amount of *Tannerella forsythia*, *Streptococcus mitis/parasanguinis*, *Filifactor alocis*, *Parvimonas micra*, and *Eubacterium saphenum* was higher in patients affected by periodontitis compared to controls. Conversely, *Veillonella sp.* and *Campylobacter concisus* were higher in salivary samples collected from healthy subjects [116].

Oral leukoplakia and recurrent aphthous stomatitis are two of the most common diseases of the oral mucosa. Oral leukoplakia is a potentially malignant disorder characterized by a white lesion generally asymptomatic that appears in the oral cavity [117], whereas the clinical presentations of recurrent aphthous stomatitis consist of very painful oral ulcers affecting 20% of the worldwide population [118]. In the last few years, an increasing number of studies have focused on the imbalance of oral microbiota and the potential relationship with these mucosal diseases. In 2017, Amer et al. [119] observed an altered microbiota in oral leukoplakia patients, where the relative abundance of *Fusobateria* was increased compared to healthy controls, while the *Firmicutes* amount was significantly reduced. Regarding recurrent aphthous stomatitis, a recent study showed that many infectious pathogens, such as *Haemophilus*, *Actinobacillus*, *Vibrio*, and *Prevotella*, were more abundant in patients affected by this mucosal disease compared to disease-free subjects, suggesting that the imbalance of oral microbiota could play a critical role in the onset of recurrent aphthous stomatitis [120].

At the same time, some studies have suggested the involvement of the oral microbial community in the development of oral cancer. For example, Zhao et al. [121] focused on oral squamous-cell carcinoma, the most frequent malignancy of the oral cavity, evaluating the role of oral microbiota in the onset of this tumor. Specifically, the researchers found that *Peptococcus*, *Fusobacterium*, *Parvimonas*, *Filifactor*, *Peptostreptococcus*, *Dialister*, and *Catonella* were enriched in the malignant lesion samples compared to controls [121]. Similarly, Sarkar and colleagues [122] detected a significant difference between cancer lesions and disease-free tissues. Interestingly, the relative abundance of *Pseudomonas*, *Corynebacterium*, *Noviherbaspirillum*, *Prevotella*, and *Deinococcus* genera was higher in oral squamous carcinoma samples, whereas genera *Serratia*, *Sutterella*, *Anoxybacillus*, *Stenotrophomonas*, and *Actinomyces* were lower compared to healthy controls [122].

Overall, the results of these studies suggested that the dysbiosis of oral microbiota may represent a triggering factor for the aforementioned diseases. However, further investigations are needed to better clarify the role of the microbial community in the onset and development of these oral diseases.

## 7. The Relationship between Oral Microbiota and OLP Development

Although OLP is considered an immunological process, the exact etiology and pathogenesis of this oral disorder remain unclear [52]. This represents the most important issue for the development of effective therapeutic and preventive strategies. Several endogenous and exogenous factors seem to be implicated in the onset and progression of OLP, such as genetic factors, viral infections, stress, depression, medications, oral hygiene, and microorganisms [12,123].

In this context, oral microbiota has recently received strong interest from the scientific community for its importance in maintaining oral homeostasis and preventing disease development [124]. Therefore, a growing body of studies is focusing on oral microbiota composition to completely understand how oral microbial changes can be related to the development of OLP.

For example, in 2016, Wang K. et al. [125] characterized the oral microbiota composition of saliva samples from OLP patients to better understand the potential relationship between the alteration of the microbial community and this oral disease. First, the research group observed that the oral microbiota of all three considered groups (erosive OLP, reticular OLP, and the control group) presented five dominant phyla (*Bacteriodetes*, *Actinobacteria*, *Fusobacteria*, *Firmicutes*, and *Proteobacteria*). However, the microbial composition was significantly different at the genus and species levels. In particular, the amount of *Solobacterium* was higher in the reticular group, while *Porphyromonas* was more abundant in the erosive OLP patients. Conversely, the relative abundance of *Campylobacter*, *Corynebacterium*, *Cellulosimicrobium*, and *Haemophilus* was increased in healthy controls compared to reticular and erosive groups. At the species level, *Prevotella melaninogenica* was more abundant in the reticular group, whereas *Streptococcus parasanguinis*, *Rothia aeria*, and *Haemophilus parainfluenzae* had lower levels in the erosive group. Furthermore, Wang K and colleagues [125] measured the levels of *interleukin* (*IL*)*-17* and *IL-23* to evaluate the potential correlation with the detected bacteria. Briefly, *Abiotrophia*, *Porphyromonas*, *Fusobacterium*, and *Paraprevotella* showed a positive correlation with salivary levels of *IL-23*, while *Parvimonas* and *Gemella* were positively related with *IL-17* levels [125]. Overall, the results of the study highlighted the differences between the oral microbiota of patients affected by OLP and healthy subjects, indicating that the imbalance of bacterial variety and specificity could play a key role in the progression and immune modulation of this oral disorder.

In the same year, Choi et al. [126] used buccal mucosa samples from OLP patients to study the involvement of bacterial communities in the inflammatory response of T cells. The in situ hybridization showed that bacterial signals were high both in the epithelium and lamina propria of OLP patients, while the controls mainly showed a high signal in the epithelium. Interestingly, the researchers also found a positive correlation between the levels of infiltrated CD3^+^, CD4^+^, and CD8^+^ cells and bacteria detected in the epithelium and lamina propria of subjects affected by this disorder. Finally, Choi and colleagues [126] evaluated the potential association between oral microbiota changes and OLP. Of note, they observed that the relative amount of *Bacteroidetes* was decreased in healthy controls compared to the OLP group, while the abundance of *Firmicutes* was increased. In addition, the amount of *Leptotrichia* and *Acinetobacter* resulted lower in the control group, whereas the relative abundance of *Streptococcus* and *Escherichia* resulted higher compared to OLP samples [126]. The findings suggested that the oral bacterial community could be involved in the etiology of OLP, damaging the epithelial barrier and favoring T-cell infiltration.

Interestingly, He et al. [127] characterized and studied the bacterial profile of buccal mucosa in OLP patients. Specifically, buccal scraping samples were collected from OLP patients, and the obtained results were compared to healthy controls. The analysis showed that several genera were detected in both groups, such as *Fusobacterium*, *Streptococcus*, *Haemophilus*, *Neisseria*, *Actinomyces*, *Prevotella*, *Leptotrichia*, and *Veillonella* [127]. However, the amount of these genera was significantly different between the two considered groups. Specifically, *Leptotrichia*, *Fusobacterium*, and *Lautropia* were found to be more abundant in OLP patients, while the abundance of *Streptococcus* was higher in the disease-free group. In addition, some bacteria were only observed in the OLP group, including *Moraxella*, *Hymenobacter*, *Mobiluncus*, and *Achromobacter* [127]. The results indicated that patients affected by OLP presented a unique bacterial profile, highlighting that the dysbiosis of oral microbiota could be implicated in the progression of this pathological condition.

In another study, biopsies of oral mucosa from OLP patients were used to evaluate cytokine levels; moreover, OLP salivary samples were used to investigate the oral microbiota profile [128]. Compared to non-specific inflammatory lesions (controls), the researchers observed that tissue samples of OLP were characterized by a larger number of infiltrated lymphocytes, especially CD4^+^ T cells. In addition, biopsies of diseased oral mucosa showed higher expression levels of *INF-γ* and *IL-33*, while only *INF-γ* was enhanced in the saliva of OLP patients compared to the disease-free group. Regarding the oral microbiome profile, the composition of salivary samples was different between the two groups. In particular, *Firmicutes* and *Actinobacteria* were more abundant in the OLP group, while the amount of *Proteobacteria* was higher in the controls [128]. At the species level, *Streptococcus oralis*, *Streptococcus salivarius*, *Streptococcus sanguinis*, *Haemophilus parainfluenza*, *Haemophilus parahaemolyticus*, and *Neisseria oralis* were mainly detected in non-specific inflammatory lesions, whereas the amount of *Fusobacterium nucleatum*, *Campylobacter rectus*, and *Neisseria mucosa* was higher in the OLP group [52]. The study highlighted the importance of the microbial community in the etiology of oral disorders, suggesting that the alteration of the oral microbiota profile could represent an innovative diagnostic biomarker for OLP identification.

Afterward, Yu et al. [129] investigated the microbial composition in the saliva of OLP patients (erosive and non-erosive form) and compared it with the microbial profile of recurrent aphthous ulcer patients and healthy controls. Specifically, they noted that *Escherichia-Shigella*, *Lactobaccilus*, and *Thauera* were three of the most abundant bacterial species found in the recurrent aphthous ulcer group. Conversely, the abundance of *Sphingomonas* and *Streptococcus* was lower compared to OLP and control groups. Interestingly, Yu and colleagues [129] also evaluated the differences in the salivary microbiome between OLP patients and healthy controls. Briefly, the authors observed that the abundance of *Oribacterium* and *Abiotrophia* was higher in the saliva of both erosive and non-erosive OLP patients than the controls, while the presence of *Aggregatibacter* was lower. In addition, the amount of *Neisseria* and *Haemophilus* was higher in the non-erosive group, whereas *Gemella* and *Lautropia* were observed especially in the erosive group [129]. The described results showed a specific microbiome profile for OLP that could be useful to differentiate the erosive from the non-erosive form of this disorder.

Similarly, Wang X et al. [130] evaluated the microbial profile in saliva and tissue samples of OLP patients. First, they observed that the microbiota composition in saliva and tissue samples of OLP patients was different. Briefly, the researchers observed that the abundance of *Escherichia-Shigella* and *Megasphaera* was higher in tissue samples, while the amount of *Capnocytophaga* and *Gemella* was higher in saliva. Moreover, the authors also found that the amount of *Carnobacteriaceae*, *Caulobacteraceae*, *Flavobacteriaceae*, and *Veillonellaceae*, as well as *Sphingomonas* and *Delftia*, was higher in both saliva and tissue samples of OLP patients compared to the control group [130]. Overall, the study suggested that environmental factors could be responsible for the differences found in the microbial profile of saliva and tissue samples of OLP patients and further confirmed that oral microbiota could play an important role in the etiology of OLP. Table 2 summarizes the most interesting results previously discussed on oral microbiota dysbiosis in OLP patients.

As emerged from the studies described above, a great heterogeneity in the microbial profiles is associated with the development of OLP. This heterogeneity mainly depends on the different sources of samples and approaches used for the profiling of both microbiota and microbiome composition. Nevertheless, some independent studies are concordant in associating *Leptotrichia* and *Lautropia* enrichment with the presence of OLP lesions [123,124,126]. Similarly, reductions in the abundance of *Streptococcus* spp. may be indicative of the presence of OLP when observed in buccal mucosa samples [123,124] (Table 2). Overall, these data indicate that the precise profiling of microbial alterations associated with the development and progression of OLP should be performed in both saliva and buccal mucosa samples. In addition, the correct evaluation of oral cavity lesions cannot ignore a careful evaluation of other molecular or morphological alterations indicative of the nature of the lesion itself and predictive of its potential neoplastic evolution.

## 8. Conclusions

OLP is a chronic inflammatory autoimmune disease of the oral cavity with an unknown etiopathogenesis. Over the years, several triggering factors have been proposed, including viral infections, medications, dental restoration materials, genetic factors, and psychological disorders. Among these exogenous and endogenous factors, the oral microbiota has recently attracted the interest of the scientific community for its importance in maintaining health status. 

While the connection between oral dysbiosis and oral lesions has been extensively studied, emerging evidence suggests a bidirectional relationship between oral diseases and alterations of the composition of both the oral microbiota and microbiome [131].

Chronic inflammation and immune dysregulation associated with both oral lichenoids and oral lichen planus may create a favorable environment for shifts in the oral microbial community. The disruption of oral mucosal integrity by lichenoid lesions could facilitate the colonization and proliferation of opportunistic pathogens or dysbiotic microbial species. Therefore, microbiota and microbiome alterations are often the consequences of the diseases and not the causes as demonstrated by changes in microbial diversity or abundance in the same patients who developed oral lesions [123,132]. Understanding how oral lichenoids and oral lichen planus influence the oral microbiota and microbiome could provide valuable insights into the pathogenesis and management of these oral diseases as well as in the identification of microbial signatures predictive of the prognosis of these lesions. Further investigation into the complex interplay between host immune responses and oral microbial communities is warranted to delineate the precise mechanisms underlying these associations.

Overall, according to the studies reported in this review article, the imbalance of the oral microbial profile may play a critical role in several oral diseases, such as dental caries, peri-implantitis, periodontal and mucosal diseases, and oral cancer. Interestingly, many studies have also investigated the potential relationship between oral bacteria and OLP, suggesting that oral microbiota dysbiosis could represent a new diagnostic biomarker for this disorder. However, the heterogeneity of the oral lesions analyzed in these papers as well as the different approaches and biological samples used for the analysis of the microbiota and microbiome composition represent key limitations that currently prevent researchers and clinicians from obtaining conclusive results on this topic.

In conclusion, the present review article could represent the starting point for the identification and validation of specific oral microbial profiles strictly associated with OLP. However, further studies are mandatory to better clarify the underlying mechanisms of action, as well as to improve prevention and develop more effective treatments against OLP.

## Figures and Tables

**Table 1:** Localization and clinical manifestations of OLP subtypes.

Pattern	Localization	Clinical Manifestations	Ref.
Reticular	buccal mucosa; tongue; gingiva; palate; lips	Wickham striae surrounded by an erythematous area	[32]
Erosive	gingiva; tongue	Keratotic white striae and ulcerations covered by a fibrin plaque	[33]
Atrophic	gingiva; tongue dorsum	Red lesions and mucosal atrophy	[34]
Plaque-like	tongue; buccal mucosa	Whitish and raised lesions	[35]
Bullous	soft palate; lips	Ulcerated areas due to the rupture of bullae	[36]
Papular	soft palate; lips	White papules in combination with other patterns	[36]

**Table 2:** The imbalance of oral microbiota in OLP patients compared to the health condition.

Sample	Microbial Profile Variations	Ref.
Saliva	↑: *Solobacterium*, *Porphyromonas* ↓: *Campylobacter*, *Corynebacterium*, *Cellulosimicrobium*, *Haemophilus*	[52]
Buccal mucosa	↑: *Leptotrichia*, *Acinetobacter* ↓: *Streptococcus*, *Escherichia*	[123]
Buccal mucosa	↑: *Leptotrichia*, *Fusobacterium*, *Lautropia* ↓: *Streptococcus*	[124]
Saliva	↑: *Firmicutes*, *Actinobacteria* ↓: *Proteobacteria*	[125]
Saliva	↑: *Oribacterium*, *Abiotrophia*, *Neisseria*, *Haemophilus*, *Gemella*, *Lautropia* ↓: *Aggregatibacter*	[126]
Saliva and tissue	↑: *Carnobacteriaceae*, *Flavobacteriaceae*, *Delftia*, *Caulobacteraceae*, *Sphingomonas, Veillonellaceae*	[127]

(↑) Increased or (↓) reduced in OLP patients compared to healthy subjects.

## Data Availability

The data reported in this manuscript are available from the corresponding author on request. The original contributions presented in this study are publicly available. These data can be found at the following: www.pubmed.com (accessed on 5 January 2024); http://www.homd.org (accessed on 29 September 2023).
